# Multiplex PCR for differentiating *Ornithodoros* tick vectors in South Africa: Proof of concept

**DOI:** 10.4102/ojvr.v92i1.2225

**Published:** 2025-10-27

**Authors:** Susan West, Juanita van Emmenes, Carin Boshoff

**Affiliations:** 1 Department of Biomedical Sciences, Faculty of Sciences, Tshwane University of Technology, Pretoria, South Africa; 2 Department of Vaccine and Diagnostic Development, Onderstepoort Veterinary Research, Agricultural Research Council, Pretoria, South Africa; 3 Department of Production Animal Studies, Faculty of Veterinary Science, University of Pretoria, Pretoria, South Africa

**Keywords:** phylogeography, African swine fever, multiplex PCR, *Ornithodoros*, ticks

## Abstract

African swine fever (ASF) is a severe viral haemorrhagic disease affecting domestic pigs, maintained by ASF virus within an ancient sylvatic cycle involving *Ornithodoros* ticks that inhabit warthog burrows. This study aimed to develop and optimise a multiplex polymerase chain reaction (PCR) assay to differentiate three *Ornithodoros* species in South Africa – *O. moubata, O. phacochoerus* and *O. waterbergensis*, to enhance ASF vector identification and management. The final multiplex PCR effectively distinguished between species with specific amplicon sizes: 180 base pairs (bp) for *O. moubata*, 640 bp for *O. phacochoerus* and 1000 bp for *O. waterbergensis*.

**Contribution:**

This refined multiplex PCR method demonstrates proof of concept as a reliable and rapid tool for distinguishing *Ornithodoros* species and correlating them with their geographical origins. This assay, based on amplification size, provides crucial information about the distribution of these species, which could enhance ASF control efforts.

## Introduction

African swine fever (ASF) is a severe viral haemorrhagic disease that affects domestic and wild pigs (Penrith 2013; Wilkinson 1989), posing a major threat to the global pig industry. The disease is caused by the African swine fever virus (ASFV), a double-stranded deoxyribonucleic acid (DNA) virus belonging to the family Asfarviridae, genus *Asfivirus* (Arias et al. 2017; Penrith, Thomson & Bastos 2004). The virus can infect a wide range of hosts, including domestic pigs, wild pigs (such as warthogs and bush pigs) and soft ticks of the genus *Ornithodoros*, which play a critical role in maintaining and transmitting the virus in some ecosystems (Bastos, Fasina & King 2014; Jori et al. 2013). Despite ongoing research, because of the complexity of the ASFV genome and virus particles, a significant delay in vaccine development is experienced (Arias et al. 2017), and control efforts are primarily focused on biosecurity, culling of infected animals and restricting the movement of pigs in affected areas (Bastos et al. 2014).

The disease originated in sub-Saharan Africa, where it circulates in an ancient sylvatic cycle between warthogs and *Ornithodoros* soft ticks. Therefore, this makes ASFV the only DNA virus transmitted by arthropods (arthropod-borne virus [Arbo]) (Blome, Franzke & Beer 2020). Since its discovery in East Africa in the 1970s, this warthog–tick sylvatic cycle of ASFV has been investigated in several geographical locations (Dixon et al. 2020; Thomson 1985). This cycle is only found in regions, mostly in East and Southern Africa, where the *Ornithodoros moubata* species complex is prevalent (Bakkes et al. 2018). Only the *O. moubata* complex has proven to be fully capable of spreading ASFV throughout Africa thus far, with vector competence seen as the physiological capacity of a vector organism to acquire, sustain and spread an infectious agent (Dixon et al. 2020). The role of these ticks in the transmission of ASFV has been confirmed through experimental infections, which have shown that different isolates of ASFV can persist in tick populations for extended periods (Burrage 2013; Kleiboeker & Scoles 2001). The ASF-controlled area, established in South Africa in 1935, encompasses the natural distribution range of warthogs, primarily in the northeastern regions of the country (De Kock, Robinson & Keppel 1940). However, the translocation of warthogs to southern areas, from 1963 onwards, driven by the expansion of wildlife farming and conservation initiatives, has led to the establishment of extralimital populations beyond their historical range (Bakkes et al. 2018; Craig et al. 2021; Swanepoel, Schulze & Cumming 2016).

A significant advancement in the study of *Ornithodoros* ticks occurred when Walton (1962) reclassified *Ornithodoros moubata*, a key vector of the ASFV, by designating a new type specimen from Groot Marico, South Africa (Craig et al. 2022). The *Ornithodoros moubata* complex was considered to include four distinct species: *O. apertus, O. compactus, O. porcinus* and the original *O. moubata* (Walton 1962), while *O. porcinus* was further divided into two subspecies (*O. p. domesticus* and *O. p. porcinus*). In South Africa, *Ornithodoros* ticks are widely distributed across regions such as Limpopo, Mpumalanga and North West provinces, which also harbour significant populations of warthogs. In 2008–2012, the distribution of *Ornithodoros* ticks in warthog burrows beyond the controlled area in North West province, Gauteng, Limpopo and Mpumalanga was described (Magadla et al. 2016). However, much of the diversity and distribution of these tick vectors across sub-Saharan Africa, particularly in their natural habitats, remains poorly understood. Researchers have uncovered geographically restricted lineages within *O. porcinus*, corresponding to different regions in Southern and Eastern Africa (Bastos et al. 2009). Further taxonomic refinement identified four previously unrecognised species and reinstated a nomen nudum, bringing the total number of Afrotropical *Ornithodoros* species to 10. In the revised taxonomy, *O. porcinus* is now confined to central East Africa, while *O. compactus, O. moubata, O. phacochoerus* and *O. waterbergensis* are confined to Southern Africa (Bakkes et al. 2018). African swine fever virus was detected in four members of the *Ornithodoros moubata* complex, including *O. waterbergensis, O. phacochoerus, O. moubata* and *O. compactus*, all of which are known to occur in South Africa (Bakkes et al. 2018; Craig et al. 2022; Jori et al. 2023; Pienaar et al. 2025). However, while *O. compactus* has been associated with tortoises as hosts, the other three species are known to parasitise warthogs (Bakkes et al. 2018; Pienaar et al. 2025). Given that warthogs play a significant role in the sylvatic cycle of ASFV in South Africa (Dixon et al. 2020; Thomson 1985), there is currently no evidence implicating tortoises in the circulation of ASFV (Bakkes et al. 2018; Craig et al. 2022; Jori et al. 2023; Pienaar et al. 2025), with definitive vector competence studies still lacking. Key taxonomic uncertainties regarding the classification of various *Ornithodoros* species continue to challenge efforts to fully comprehend their role in ASF epidemiology. The ability of ASFV to infect *Ornithodoros* ticks varies significantly depending on the virus isolate and the origin of the ticks (Craig et al. 2022). Experimental infections of *Ornithodoros* ticks have been conducted to assess the maintenance and transmission of ASFV (Ribeiro et al. 2015). However, the diversity and distribution of vectors in natural populations throughout the sylvatic cycle in sub-Saharan Africa remain understudied, and key taxonomic uncertainties persist (Boshoff et al. 2014).

The Argasidae family, particularly the *Ornithodoros* genus, is highly diverse, and the existence of cryptic species (species that are morphologically similar but genetically distinct) further complicates classification (Mans et al. 2019). While molecular data are crucial for resolving taxonomic uncertainties, they are not available for all *Ornithodoros* species. This lack of genetic information hinders the ability to assess morphological variability within and between species and to define generic arrangements (Mans et al. 2019). There are ongoing debates about the placement of various subgenera within the *Ornithodoros* genus, with molecular data being needed to clarify these relationships (Chen & Lui 2022). These factors contribute to disagreements on species boundaries and relationships between genera, particularly at the subgenus level (Mans et al. 2019).

Currently, no molecular assays using a multiplex approach are available for identifying different *Ornithodoros* species. This study presents a proof of concept for a multiplex technique aimed at differentiating *Ornithodoros* tick species associated with warthogs (*O. moubata, O. waterbergensis* and *O. phacochoerus*), which are implicated in the sylvatic cycle within the historical warthog distribution range in South Africa. Establishing such a method lays the groundwork for future applications in tracking the spread of ASF and inferring the geographic origin of the vector – information that is vital for enhancing disease surveillance and control strategies.

## Research methods and design

### Tick homogenates and deoxyribonucleic acid extraction

Ticks were collected from 11 different game parks and nature reserves in South Africa, including Gauteng, North West and Limpopo provinces (C.I. Boshoff [Tshwane University of Technology], pers. comm., 01 February 2019), as well as from two game reserves in the Kingdom of Eswatini (formerly known as Swaziland) (Boshoff et al. 2014). Eswatini was included in the study because of its location between the KwaZulu-Natal and Mpumalanga provinces of South Africa, within the ASF-controlled area. The country is also known for the presence of game parks and warthog populations, which are relevant to the study. The ticks were individually collected during 2012–2013 as part of a previous study (C.I. Boshoff [Tshwane University of Technology], pers. comm., 01 February 2019 and Boshoff et al. 2014). Field teams used established collection methods, which involved manually extracting ticks from warthog burrows – one of the primary habitats of *Ornithodoros* ticks in Southern Africa. Each tick was carefully handled to prevent contamination and was then processed for analysis. This method of collection is essential for ensuring that the tick samples are representative of natural populations, particularly in regions where the sylvatic cycle of ASF is prevalent. Ticks were homogenised as described by Boshoff et al. (2014) and stored at –20 °C at the Transboundary Animal Diseases – Onderstepoort Veterinary Research (TAD-OVR) biosafety level 3 (BSL3) laboratories, Agricultural Research Council. The 40 samples selected for this study were chosen based on their geographic origin to represent different tick populations from across the regions sampled. The selected samples originated from 29 burrows, ensuring a wide distribution of ticks within and across the different provinces and game parks, as well as nature reserves. Deoxyribonucleic acid was extracted from the stored tick homogenates using the high pure polymerase chain reaction (PCR) template preparation kit (Roche Diagnostics GmbH, Germany), following the manufacturer’s instructions.

### 16S Ribosomal RNA amplification and sequencing

For the present study, a final set of 40 tick samples was selected from the larger collection of 212 homogenates, which were tested for positive amplification of the 16S ribosomal ribonucleic acid (rRNA) gene. To verify the integrity of the DNA, PCR was conducted using the extracted DNA from the 40 selected homogenates to amplify the 16S rRNA region of *Ornithodoros* ticks. Primers targeting highly conserved regions within the aligned mitochondrial 16S rRNA gene were used, as previously described by Bastos et al. (2009). The DNA amplicon products generated from the 16S PCR were subsequently purified to prepare the final PCR product. This purified product, along with at least one primer (16S-FArg) at the appropriate concentration (0.5 µM), was used for the sequencing reaction on the 3500XL Genetic Analyzer (Applied Biosystems™, United States [US]). The resulting nucleotide sequences were aligned and compared with partial 16S rRNA data representing *Ornithodoros* species available in GenBank *O. moubata* (NC004357; AB073679), *O. waterbergensis* (KR907251; KJ133593) and *O. phacochoerus* (KJ133596; KJ133597), to assist with groupings (data not shown).

### Multiplex polymerase chain reaction development: Species-specific primer design

**FIGURE 1:**
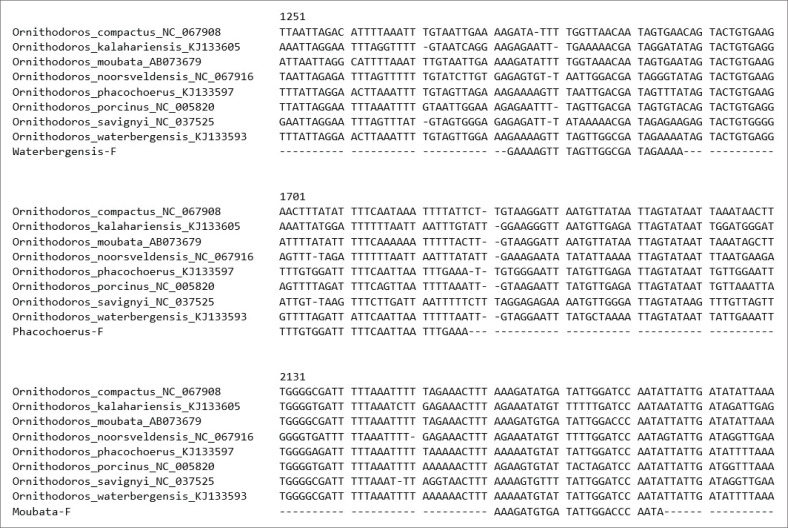
Alignments of ribosomal ribonucleic acid *Ornithodoros* dataset, showing primer placements, with positions indicated against NC067916.

The primer design strategy involved using the published 16S-RArg primer as the reverse primer, as described by Bastos et al. (2009), and creating forward primers specific to each of the three species. Forward primers were designed using Qiagen CLC Main Workbench 6.0 (CLC bio, Qiagen, Germany) to work in conjunction with the 16S-RArg primer (Boshoff et al. 2014) for detecting the unique regions of the three species described by Bakkes et al. (2018). The primers were developed based on the alignment of complete rRNA *Ornithodoros* genomes representing *O. moubata* (NC004357; AB073679), *O. waterbergensis* (KR907251; KJ133593) and *O. phacochoerus* (KJ133596; KJ133597) to establish zones with variability or similarities, which are available from GenBank (Figure 1). Table 1 provides detailed information on the primer sequences for each species and the approximate amplicon size.

**TABLE 1:** Sequence and amplicon size of three different developed species-specific primers of *Ornithodoros* species occurring in South Africa.

Primer name	Primer sequence	T_m_ (°C)	Amplicon size*(bp)	Reference
16S-RArg	5′-CCGGTCTGAACTCAGATCA-3′	57	-	Bastos et al. (2009)
Moubata-F	5′-AAAGATGTGATATTGGACCCAATA-3′	57	180	This study
Phacochoerus-F	5′-TTTGTGGATTTTTCAATTAATTTGAAA-3′	56	640	This study
Waterbergensis-F	5′-GAAAAGTTTAGTTGGCGATAGAAAA-3′	57	1000	This study

*Source:* West, A.S., 2020, ‘Phylogeography of the *Ornithodoros* vector of African Swine Fever in South Africa’, Master’s thesis, Tshwane University of Technology, Pretoria.

bp, base pairs; RArg, retinoic acid receptor gamma.

Note: Please see the full reference list of the article, https://doi.org/10.4102/ojvr.v92i1.2225, for more information.

Optimal parameters for the primer design were maintained, including a melting temperature (T_m_) between 48 °C and 58 °C, a primer length of 20 base pairs (bp) to 28 bp, a GC content of 40% – 60%, a maximum 3’ stability of 8.0 kcal/mol and a maximum self-complementarity of 18.0 (Shen et al. 2010). All primer sequences were screened using the Basic Local Alignment Search Tool (BLAST) to ensure specificity by comparing them against available databases. The primers were designed to target conserved regions within the aligned mitochondrial 16S rRNA, resulting in species-specific differences in amplicon size. Variation in amplicon size was considered to produce amplicons ranging from 200 bp to 1000 bp, ensuring compatibility with conventional PCR and Taq polymerase.

### Uniplex polymerase chain reaction evaluation of the developed primers

All samples were initially tested using primers targeting the conserved regions of the mitochondrial 16S rRNA gene of *Ornithodoros* ticks to assess nucleic acid integrity (Table 2). Each primer pair was individually tested on samples representing the three species. The uniplex PCR reaction for *O. moubata, O. phacochoerus* and *O. waterbergensis* was conducted in a 50 µL reaction mixture containing 25 µL of Promega GoTaq^®^ G2 hot start green master mix (Promega M7422, US), 1.0 µL of each forward primer at a concentration of 0.5 µM (Table 1), 1.0 µL of 0.5 µM 16S-RArg (retinoic acid receptor gamma) primer, 15 µL of nuclease-free water (Promega P1193, US) and 5 µL of DNA template. In the negative control reaction, nuclease-free water was used instead of soft tick DNA. Amplifications were performed in a MiniAmp™ Thermal Cycler (Applied Biosystems™, US) under the following conditions: initial denaturation at 96 °C for 12 min, followed by 40 cycles of 96 °C for 30 s, 51 °C for 30 s and 70 °C for 1 min, with a final extension at 70° C for 5 min. The resulting amplicons were analysed by electrophoresis on a 1.5% agarose gel (Agarose I™, VWR Life Science, US), stained with 0.2 mg/mL ethidium bromide (Promega H5041, US) and compared against a 100 bp ladder (Promega G2101, US).

**TABLE 2:** Summary of 40 tick partial 16S ribosomal ribonucleic acid sequences: Classification by *Ornithodoros* species and species-specific primers, including tick count and locations.

Tick species	16S rRNA sequences (tick count)	Species-specific PCR (tick count)	Province, country (locations)
*O. moubata*	14	14	Gauteng, South Africa; North West, South Africa
*O. phacochoerus*	13	13	Mpumalanga, South Africa; eSwatini
*O. waterbergensis*	13	13	Limpopo, South Africa

*Source:* West, A.S., 2020, ‘Phylogeography of the *Ornithodoros* vector of African Swine Fever in South Africa’, Master’s thesis, Tshwane University of Technology, Pretoria.

rRNA, ribosomal ribonucleic acid; PCR, polymerase chain reaction.

### Optimisation of the multiplex polymerase chain reaction conditions

The multiplex PCR utilised the Moubata-F, Phacochoerus-F, Waterbergensis-F and 16S-RArg primers (Table 1). To optimise the amplification of *O. waterbergensis* and *O. phacochoerus*, the primer concentrations were tested at 1.5 µM, 2 µM and 2.5 µM. The reverse primer was consistently used at a concentration of 3.0 µM to ensure adequate binding with all three forward primers.

To evaluate the specificity of the multiplex PCR, representative samples from each species were tested. Subsequently, the optimal annealing temperature was determined by testing at 51 °C, 55 °C and 59 °C. The melting temperatures (T_m_) for the three forward primers, 57 °C, 56 °C and 57 °C (Table 1), were used as references to select the annealing temperatures (Ta = T_m_ – 5 °C) for this optimisation process.

### Multiplex polymerase chain reaction

After optimisation, the multiplex PCR was carried out in a 50 µL reaction mixture containing 10 µL of MyTaq reaction buffer (Bioline, A Meridian Life Science^®^ Company, Celtic Molecular Diagnostics), 1.5 µM of all-F primers, 3.0 µM 16S-RArg primer, 15 µL of nuclease-free water (Promega P1193, US) and 5 µL of DNA template. Nuclease-free water was used in place of soft tick DNA for the negative control reaction. Amplifications were performed using a MiniAmp™ Thermal Cycler (Applied Biosystems™, US) under the following conditions: initial denaturation at 96 °C for 12 min, followed by 40 cycles of 96 °C for 30 s, 51 °C for 30 s and 70 °C for 1 min, with a final extension at 70 °C for 5 min. The resulting amplicons were analysed by electrophoresis on a 1.5% agarose gel (Agarose I™, VWR Life Science, US), stained with 0.2 mg/mL ethidium bromide (Promega H5041, US) and compared against a 100 bp ladder (Promega G2101, US).

### Ethical considerations

Ethical clearance to conduct this study was obtained from Onderstepoort Veterinary Institute Animal Ethics Committee (OV24/01/P001) and a permission letter from Agriculture Forestry & Fisheries (12/11/1/1/14).

## Results

### 16S Ribosomal ribonucleic acid tick gene amplification

**FIGURE 2:**
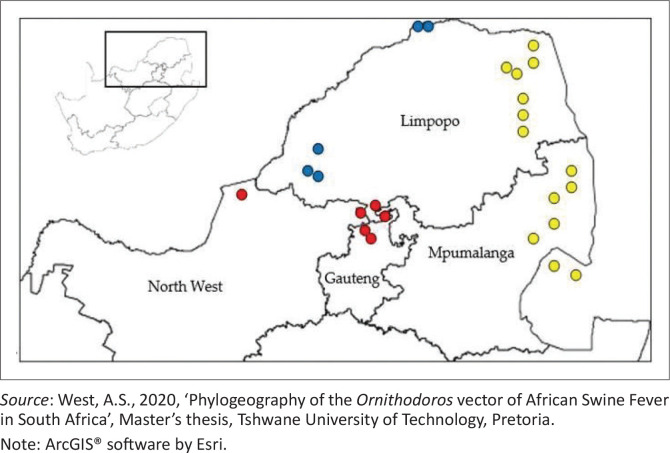
Map showing the burrow location of the ticks tested in the multiplex polymerase chain reaction, indicating the identified species distribution: *O. moubata (●[red]), O. phacochoerus (●[yellow])* and *O. waterbergensis (●[blue])*.

A total number of 40 samples met the criteria of positive amplification for the 16S rRNA gene at 313 bp, representing the corresponding regions and were used for multiplex PCR development. The selected samples provided a diverse representation across the different parks and/or reserves and species in question. Genebank comparison analysis using partial 16S rRNA, including *O. moubata* (NC004357; AB073679), *O. waterbergensis* (KR907251; KJ133593) and *O. phacochoerus* (KJ133596; KJ133597) as the reference sequences, revealed the distribution of *Ornithodoros: O. moubata, O. phacochoerus* and *O. waterbergensis* (Table 2). These species correspond to the clades and the species classification by Bakkes et al. (2018). The *O. phacochoerus* group formed a distinct clade in the Kruger National Park, Limpopo province. The *O. waterbergensis* group grouped phylogenetically with ticks from Marakele and Mapungubwe. The *O. moubata* group included ticks from Dinokeng and Madikwe, indicating their representation in the Gauteng and North West provinces (Figure 2).

### Uniplex polymerase chain reaction

**FIGURE 3:**
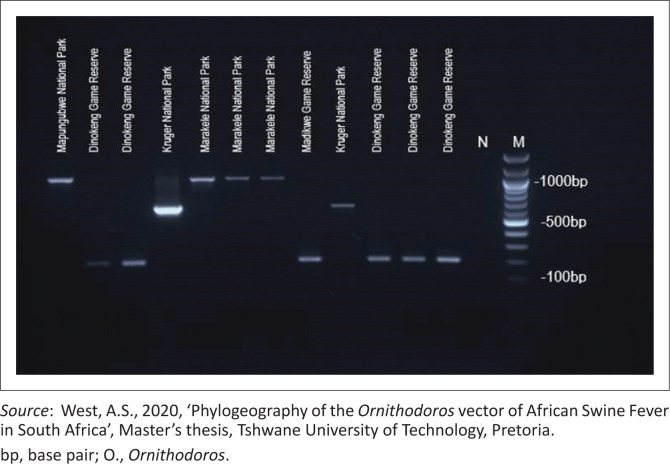
Agarose gel electrophoresis of the polymerase chain reaction products to confirm *Ornithodoros* species from different geographical samples (180 bp for *O. moubata* [lanes 2, 3, 8 and 10–12], 1000 bp for *O. waterbergensis* [lanes 1 and 5–7] and 640 bp for *O. phacochoerus* [lanes 4 and 9]). A 100-bp marker in lane M (Promega) and nuclease-free water in lane N.

Uniplex PCR using specific primers for each species confirmed the distinct amplification sizes for *O. moubata* (180 bp), *O. phacochoerus* (640 bp) and *O. waterbergensis* (1000 bp), as indicated in Figure 3 and Table 2. This allowed for effective differentiation between the species based on the PCR product size. The samples originating from Limpopo province matched the 1000-bp size of *O. waterbergensis* (Mapungbukwe and Marakele parks), samples from Gauteng matched the 180-bp size of *O. moubata* (various areas within Dinokeng Game Reserve) and samples from KNP matched the 640-bp size of *O. phacochoerus*. The results were expected to correlate with the area of origin and are comparable to the findings of Bakkes et al. (2018) and Craig et al. (2022).

### Multiplex polymerase chain reaction

**FIGURE 4:**
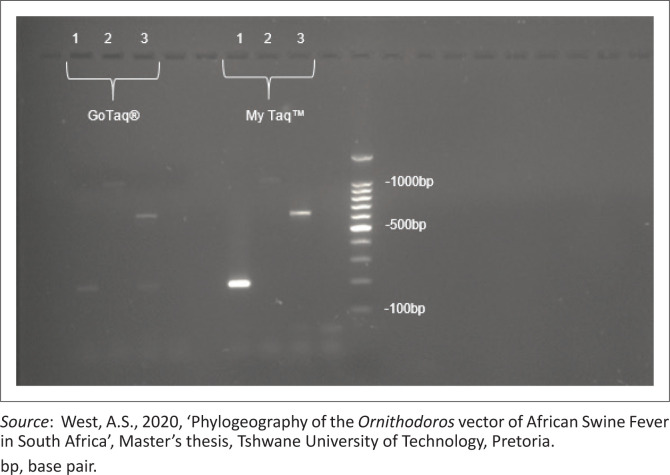
Multiplex polymerase chain reaction results with two different enzymes, Promega GoTaq^®^ and Bioline MyTaq™ HS deoxyribonucleic acid polymerase, with samples representing 180 bp, *O. moubata* (lane 1); 1000 bp, *O. waterbergensis* (lane 2) and 640 bp and *O. phacochoerus* (lane 3). 100 bp marker. Band sizes were estimated according to 100 bp molecular weight marker.

Initial multiplex PCR tests did not yield the expected bands for *O. waterbergensis* and *O. phacochoerus* at the anticipated sizes, with only the 310 bp 16S rRNA band visible. Increasing the annealing temperature to 55 °C did not resolve the issue, resulting in similar non-specific amplification problems. Subsequent use of Bioline MyTaq™ HS DNA polymerase improved results although the intensity of some amplicons remained suboptimal (Figure 4). Optimisation of primer concentrations and annealing temperatures identified 1.5 pM/µL as optimal for *O. waterbergensis* and *O. phacochoerus*, and 51 °C as the best annealing temperature (results not shown). Validation with known samples confirmed the multiplex PCR’s ability to distinguish between the species.

## Discussion

Accurate identification of ticks at the species level is essential for several reasons, including distribution, life cycle and host range and/or preference, particularly for species within the genus *Ornithodoros* that serve as biological vectors of ASFV (Jori et al. 2023). The geographical differentiation between the ticks indicates taxonomic species-specific adaptations potentially driven by the diverse environmental conditions across different habitats, such as the cracks and crevices of rustic human dwellings and livestock shelters. Such ecological insights are crucial for understanding the vector dynamics and the role of these ticks as ASFV reservoirs within their respective ecosystems, as highlighted by a recent review emphasising the need for comprehensive knowledge of *Ornithodoros* tick biology in sub-Saharan Africa (Jori et al. 2023).

As indicated in Table 2, the ticks were first screened using the universal 16S primers to confirm amplification of the tick DNA. The selected ticks were then evaluated using the newly designed primers (Table 1). The successful application of uniplex PCR in distinguishing between the three *Ornithodoros* species, *O. moubata* (180 bp), *O. phacochoerus* (640 bp) and *O. waterbergensis* (1000 bp) demonstrates the effectiveness of the primers designed for this purpose. The distinct amplification profiles validate the precision of the primers in targeting species-specific sequences, confirming the reliability and specificity of the method. In contrast, the initial multiplex PCR efforts using GoTaq encountered challenges because of non-specific amplifications and unclear band results (Figure 4). The low annealing temperatures were identified as a significant factor contributing to these issues. Because of the non-specific amplification observed with GoTaq, a different polymerase was selected for optimisation. The optimisation process, including the transition to Bioline MyTaq™ HS DNA polymerase, demonstrates the importance of polymerase selection in achieving improved specificity, yield, speed and robustness in multiplex systems. MyTaq DNA polymerase is an excellent choice for multiplex PCR, with its hot-start feature avoiding non-specific amplification and enhancing specificity across various primer sets. The final optimised conditions, including the adjusted primer concentrations and annealing temperatures, enabled the development of a functional multiplex PCR system that accurately identified the *Ornithodoros* species and aligned with their geographical origins. However, it is important to emphasise that this study represents a proof of concept, demonstrating the feasibility of the approach rather than a fully optimised diagnostic tool. For regular diagnostic applications, the existing technology has to be improved as it currently requires the use of a particular polymerase (Bioline MyTaqTM HS DNA polymerase) and yields results of varying quality. Although the morphological identification of *Ornithodoros* species is challenging because of their taxonomic complexity, this development represents a significant advancement. Further advancements are needed before this technique can be implemented in field research or routine monitoring programmes. The improved multiplex PCR methodology, while representing a valuable proof of concept, has potential implications for advancing control and management strategies for ASF in South Africa once fully optimised. Current knowledge of *Ornithodoros* ticks as reservoirs and biological vectors of ASFV in Africa is limited (Anholt et al. 2023), making the molecular methods provided in this study critical for epidemiological investigations. The vector competence of different *Ornithodoros* species varies (Pereira De Oliveira et al. 2020), with some Afrotropical soft ticks well established as ASFV vectors, while others remain poorly understood. Although improved knowledge of the distribution and ecological significance of these ticks in ASFV transmission cycles will be made possible by the capacity to quickly and correctly identify species, the limitations of the current approach must be addressed before broad use can occur.

However, further research is required to fully delineate the distribution of these tick species across Southern Africa. The current sampling provides valuable insights, but a more extensive sampling across provinces in South Africa, such as North West, Limpopo, Gauteng, Mpumalanga, Northern Cape and Free State, is necessary. These regions are not only ecologically diverse but also include both ASF control areas and regions that may be at high risk of ASF introduction or spread. A more comprehensive understanding of tick distribution in these areas would enable a more precise mapping of ASF risk zones, allowing for better-targeted control measures.

Future research should focus on understanding the specific tick–virus relationships for each of the *Ornithodoros* species identified in the study. It is well known that ASFV transmission dynamics can vary depending on the tick species involved, but the precise mechanisms underlying these differences are not yet fully understood. Investigating the vector competence of each species would provide critical insights into ASF epidemiology. This could include laboratory experiments designed to assess the vector competence of each species, as well as field studies monitoring the prevalence of ASFV in tick populations across different regions.

## Conclusion and future research

This study provides a crucial framework for distinguishing *Ornithodoros* tick species in South Africa and for assessing the geographical variation within these tick populations. *Ornithodoros* ticks are the primary vectors of ASFV in the region’s sylvatic cycle, making it essential to accurately differentiate between the species involved. The identification of these species is a critical step towards improving the understanding of ASF transmission dynamics, particularly in areas where both wild and domestic pig populations are at risk.

To address this, a multiplex PCR procedure was developed as part of the study. This method enables the rapid and reliable screening of *Ornithodoros* species, specifically linked to warthogs, to distinguish among the three species described by Bakkes et al. (2018), which are found in warthog historical areas in South Africa – *O. moubata, O. waterbergensis* and *O. phacochoerus*. The development of the multiplex PCR method was the key achievement of the study. Using the 16S rRNA gene as a genetic marker, the assay was both sensitive and specific for identifying *Ornithodoros* species. A future study is recommended to explore the development of species-specific primers for all *Ornithodoros* species around the world, further enhancing the specificity and applicability of the assay for other countries. If one would like to include this test as a diagnostic test, a full validation will be required. Future optimisation should also aim to standardise the quantification techniques and enhance the multiplex PCR reproducibility by using known DNA concentrations for every tick sample. Critical enhancements in the methodology are essential to reduce the reliance on particular polymerases and to improve the overall quality and consistency of the results. To obtain clearer, more repeatable amplification profiles, this may entail reworking the primers to function well with common polymerases, improving the buffer conditions and refining the cycling parameters. One of the most important applications of this multiplex PCR method is its potential to aid in identifying the geographical origins of ASF vectors. Understanding the distribution of specific *Ornithodoros* species can inform strategies for disease control, especially since different species may play different ecological roles and capacities for transmitting ASFV. This geographic information is essential for the effective management of ASF, particularly in regions where control efforts are focused on containing the disease within wildlife populations to prevent spillover into domestic pig herds.
